# Account of the Dissection of a Morbid Body

**Published:** 1808-02

**Authors:** H. Wadsworth

**Affiliations:** Farmington, in America


*Account of the Dissection of a morbid Body.* By Dr. H. Wadsworth, *of Farmington, in America, in a Letter to* Dr. Coxe, *dated March* 18*th*, 1806.

On the 19th of December 1805, I received a line from Southington, from my father, requesting me to assist him in dissecting the body of Ithamar Curtiss, who died the day before. I accordingly attended immediately. From its having been a tedious case, my father was unable to give me a more particular account than the following.

" I. C. of Southington, by trade a hatter, but had for several months, previous to his illness, been peddling tin in the state of Rhode-Island, applied to me on the 13th October 1804, for advice; complained of a dull pain in the head, great thirst, some uneasiness about the region of the stomach, and had been rather costive for some time; his stools of a light colour, and his appetite then very good. The patient attributed his complaints to having, while in Rhode-sland, eaten no other bread than Indian. There was no evidence of any active disorder of the liver, as he had no pain in the region of this organ or in the shoulder; neither did it appear there was any obstruction of the common duct, as there was no regurgitation of bile. From these these symptoms I concluded a deficient secretion of bile to be the cause of his disorder. Emetics, from their mechanical operation, I thought might rouse the liver from its state of torpor. Their operation was attended with essential relief of the head and stomach, and he required less physic for a few days after their operation. During the operation of emetics (of which he took several) he threw off a great quantity of viscid, acid matter, and appeared for a while to be rather convalescing: but this continued only a short time; his appetite grew voracious, and he began to emaciate and lose strength very rapidly; he had not the least pain in any part; costiveness continued I then thought proper to administer calomel in alterative doses; which were continued several weeks without any effect upon the system. The last four months of his life, he ate as much as five common healthy labourers, and drank proportionally. This enormous quantity was perfectly digested without the aid of exercise, as his strength would not admit of it; he never appeared to suffer the least inconvenience from his food, and usually slept quietly immediately after eating. His appetite continued until the 16th of December, when it failed at once; he complained of no severe pain, but universal oppression; which continued but a few hours, when he appeared to be sinking, grew comatose, senseless, and died on the morning of the 18th of December 1805."

The next morning I performed the dissection, in the presence of several physicians from the neighbouring towns. On opening the abdomen, I found the liver to occupy so great a part of the cavity, that I was unable to examine the other viscera, until I had dissected it out. I found the upper part of it much thicker than it is found when in a healthy state, and the membranes immediately investing it, of a very dark purple; and it weighed six pounds and six ounces; on cutting into it, it appeared firm and of a healthy colour. The gall-bladder was completely empty and collapsed; the passage from it to the duodenum was free from any obstruction.

I then proceeded to examine the stomach, which was empty, and so contracted, that it very little exceeded the duodenum in size. On laying it open, I found none of the rugæ of the villous coat, but a smooth surface of light yellow mucus (not pus). On scraping it with the back of the scalpel, this mucus was very easily removed, leaving bare the muscular coat, which, even in that contracted state, was not thicker than the finest glove leather. The intestines, except the colon, contained nothing but flatus The colon was so completely filled with sybalic fæces, that it was nearly three inches in diameter.

The spleen was contracted and wrinkled, so that it was not more than two inches and a half in length; and its weight could not have exceeded two ounces.

The left kidney was about three times the size of the right; which last was of the natural size.

There was a complete adhesion of the whole surface of the left lobe of the lungs, to the pleura and mediaistnum, with this singularity, that he never experienced the least degree of pneumonic affection in the course of his life.

Does the above case, in which there was so strong evidence of deficiency of bile, countenance the idea that the gastric juice, according to the doctrine of Spallanzani and Stevens, is sufficient, without the aid of the bile, to perform digestion? That digestion in this case was performed solely by the power of the gastric juice, appears probable: from its having exerted a solvent power even upon the stomach itself; at the same time exciting an appetite incompatible with the action of a mere caustic.

Considering the enlarged state of the kidney, the rapid and extreme emaciation, notwithstanding the enormous quantity of food taken and digested; it is not impossible that this case might have been connected with a diabetes mellitus; and I regret that I am not authorised to reject or confirm this supposition, by acquaintance with the state of the urinary secretion.

